# When Do Service Employees Suffer More from Job Insecurity? The Moderating Role of Coworker and Customer Incivility

**DOI:** 10.3390/ijerph16071298

**Published:** 2019-04-11

**Authors:** Yuhyung Shin, Won-Moo Hur

**Affiliations:** 1 School of Business, Hanyang University, 17 Haengdang-dong, Seongdong-gu, Seoul 133-791, Korea; yuhyung@hanyang.ac.kr; 2 College of Business Administration, Inha University, 100 Inha-ro, Michuhol-gu, Incheon 22212, Korea

**Keywords:** job insecurity, emotional exhaustion, job performance, coworker incivility, customer incivility

## Abstract

The present study examines the effect of service employees’ job insecurity on job performance through emotional exhaustion. We identified workplace incivility (i.e., coworker and customer incivility) as a boundary condition that strengthens the positive relationship between job insecurity and emotional exhaustion. To test this moderating effect, we collected online panel surveys from 264 Korean service employees at two time points three months apart. As predicted, the positive relationship between job insecurity and job performance was partially mediated by emotional exhaustion. Of the two forms of workplace incivility, only coworker incivility exerted a significant moderating effect on the job insecurity–emotional exhaustion relationship, such that this relationship was more pronounced when service employees experienced a high level of coworker incivility than when coworker incivility was low. Coworker incivility further moderated the indirect effect of job insecurity on job performance through emotional exhaustion. These findings have theoretical implications for job insecurity research and managerial implications for practitioners.

## 1. Introduction

The long-lasting recession and unpredictable changes in the global economy have exposed employees to job insecurity [1,2]. Job insecurity is defined as “the level of uncertainty a person feels in relation to his or her job continuity” [2], (p. 1249). Prior research has examined the job insecurity of employees who work in various occupations and industries [3] and has developed a model of job insecurity that is applicable across different jobs [4]. Given that job insecurity has become a critical issue for service employees [5,6], it is surprising how little research has addressed the job insecurity of such employees. To fill this research gap, our study seeks to develop and test a model of job insecurity specifically targeting service employees.

We contend that it is pivotal to examine job insecurity in the service sector for two reasons. First, frontline service employees experience a high level of work intensity and emotional labor [7,8], as well as temporary employment and minimum wage [9], which renders them fearful of losing their jobs [5]. Second, as recent technological advances such as self-service technology, artificial intelligence, automation robots, and smart devices are replacing service employees’ jobs [10], their perceived job insecurity steadily increasing. Therefore, investigating service employees’ job insecurity and the outcomes of this state is an important and timely research agenda [5,6]. In response to this call, our study aims to assess how service employees’ job insecurity affects their job performance. More specifically, drawing on affective events theory (AET [11]) and conservation of resources (COR) theory [12]—suggesting that negative work experiences elicit negative affective reactions and drain resources necessary for job-related activities, which in turn undermines job performance—we propose emotional exhaustion as a key intermediary mechanism linking job insecurity and the performance of service employees. 

AET and COR theory further posit that there are boundary conditions that strengthen or weaken employees’ reactions to negative work events. Job insecurity research has generally identified social support, job characteristics, and individual differences as moderators that interact with job insecurity [13]. In particular, prior research on job insecurity has mainly attended to moderators that can mitigate the negative effects of job insecurity (see References [5,13,14,15,16]). However, it is also necessary to explore moderators that can aggravate the negative effects of job insecurity so as to prevent or reduce negative outcomes [17]. We argue that coworker and customer incivility is of particular importance in service encounters [18]. Coworker and customer incivility refer to uncivil behavior (e.g., sarcastic, rude, or hostile verbal or non-verbal behavior) instigated by coworkers and customers, respectively [19]. Because the interpersonal relationships involved in teamwork and relationships with customers are a crucial precondition for providing high-quality service for customers [20,21], negative encounters with coworkers and customers are presumed to aggravate the deleterious effect of service employees’ job insecurity on work outcomes [22,23,24]. Thus, the second purpose of our study was to test the moderating effect of coworker and customer incivility on the relationship between service employees’ job insecurity and performance.

## 2. Theoretical Background and Hypothesis Development

### 2.1. Mediation of Emotional Exhaustion on the Job Insecurity—Job Performance Relationship

Emotional exhaustion refers to the extent to which employees feel exhausted in their job due to the depletion of their emotional resources [25,26]. The positive association between job insecurity and emotional exhaustion has been well established in the job insecurity literature (see References [27,28,29,30]). While it has been documented that job insecurity exerts a detrimental effect on job performance by aggravating psychosomatic strain [31], the mediated relationship between job insecurity, emotional exhaustion, and the job performance of service employees has rarely been tested. Given that service employees are faced with severe emotional demands arising from job-insecurity threats and difficult customer encounters, it is crucial to determine whether the job insecurity–emotional exhaustion–job performance linkage can be replicated in the service context. Thus, we identified emotional exhaustion as a central mediating process through which service employees’ job insecurity leads to impaired job performance.

While it is plausible that poor job performance renders employees vulnerable to job-insecurity and that a high level of emotional exhaustion causes them to feel insecure in their job, we claim that the causal relationship of job insecurity to negative job performance stemming from emotional exhaustion stands on stronger theoretical underpinnings than its reverse causality. The proposed mediating effect can be explicated by AET, which holds that workplace events shape affective reactions, which in turn lead to behavioral responses [11]. According to the job insecurity literature, job insecurity is a major negative event in the workplace. When service employees encounter threats to their job security, they experience negative affective states such as low levels of emotional energy and vigor [31] and high levels of anxiety, helplessness, and hopelessness. The increased levels of emotional exhaustion affect the resulting behavioral responses of service employees in a way that negatively affects their work, thereby decreasing their job performance.

COR theory also presents a theoretical account of the mediating effect of emotional exhaustion on the relationship between job insecurity and performance. The basic premise of COR theory is that individuals strive to preserve and protect their valued resources [12]. COR theory further advocates that individuals who possess sufficient resources are more capable of gaining resources, whereas those who lack resources are vulnerable to future resource losses. COR theorists identify job security as a key job stressor that drains employees’ resources [12]. In situations of job-insecurity, service employees exert extra effort in preventing further resource loss (e.g., job loss or disconnection from coworkers) rather than in acquiring additional resources [32], which leads to exhaustion of mental and emotional energy [33]. The depletion of work-related energy is particularly detrimental to service employees, who need to manage their emotions and display positive emotions even in difficult customer encounters [34]. Thus, we argue that because service employees facing job insecurity exert extra efforts to cope with job related stressors, they are emotionally exhausted and lack the necessary resources to provide customers with high-quality service. This line of reasoning lead to the following mediation hypothesis:

**Hypothesis** **1.** *The relationship between job insecurity and job performance is mediated by emotional exhaustion.*

### 2.2. Moderation of Coworker and Customer Incivility on the Job Insecurity–Emotional Exhaustion Relationship

Building on AET and COR theory, we proposed a positive relationship between job insecurity and emotional exhaustion for service employees. We further predicted that incivility instigated by coworkers and customers serves as a critical boundary condition that amplifies the positive link between job insecurity and emotional exhaustion in service contexts. Workplace incivility refers to “low-intensity deviant workplace behavior with an ambiguous intent to harm” [22] (p. 457). Examples of workplace civility include ignoring the target’s greeting, using offensive nicknames, making demeaning or sarcastic comments about the target, and not giving credit when the target deserves credit [35]. While employees experience incivility from various sources (e.g., coworkers, supervisors, workgroups, and customers) [36,37], drawing on prior findings that coworker incivility and customer incivility jointly affect employee outcomes (see References [24,34]) and the contention [18] that the two forms of incivility are of particular importance to service employees’ attitudes and behavior, we isolated coworker incivility and customer incivility as moderators that strengthen the effects of emotional exhaustion in service contexts. 

AET theorizes that contextual variables serve as boundary conditions that moderate the effects of workplace events on an employee’s affective response [11]. Given that individuals overestimate the occurrence of negative events [38], a service employee’s negative emotions are likely to become aggravated when a negative work event (e.g., job insecurity) is coupled with another negative event (e.g., coworker incivility). More precisely, we anticipate that service employees’ job insecurity has a more deleterious effect on their emotional state when their coworkers are uncivil toward them. Service employees need assistance and information to overcome threats of job insecurity. To deal with uncertainty in the workplace, they need to validate their perceptions and interpretations of job insecurity and seek methods of coping with situations of job-insecurity by communicating with their coworkers. However, uncivil behavior committed by coworkers inculcates a feeling of rejection, which prevents service employees from interacting with coworkers [18]. As a result, service employees facing coworker incivility suffer more from job insecurity and, thus, are likely to drain their emotional resources.

According to COR theory, positive social interactions can expand the reservoir of resources [12]. The COR literature suggests that employees are better able to cope with work stressors if they are equipped with social resources [12,16,39]. Put differently, employees can acquire the resources necessary for coping with work stressors if they receive support and assistance from coworkers. In contrast, employees whose resources are drained are vulnerable to future resource loss [12]. Thus, service employees who interact with uncivil coworkers are likely to remain helpless and exhausted when facing job insecurity. This is because uncivil coworkers wear out service employees’ emotional and cognitive resources [40] by violating social norms of respect and support [22]. In the COR framework, coworker incivility is a major social stressor that can drain the victim’s personal resources [18,24,34,41]. In particular, when employees desperately need resources for reducing threats to job continuity, incivility perpetrated by coworkers depletes those resources, which elicits a burnout state [42]. Therefore, we reasoned that coworker incivility interacts with job insecurity in a way that boosts the positive effects of job insecurity on emotional exhaustion.

**Hypothesis** **2.** *The relationship between job insecurity and emotional exhaustion is moderated by coworker incivility, such that the positive relationship between job insecurity and emotional exhaustion is more pronounced when coworker incivility is high than when it is low.*

Parallel to coworker incivility, we postulated the moderating effect of customer incivility based on AET and COR theory. First, building on the AET proposition that the negative effects of a negative work event on affective responses is facilitated in the presence of another negative event, we predicted that unpleasant customer encounters exacerbate the negative effects of service employees’ job insecurity. Grandey and colleagues [23] reported that employees dealing with uncivil customers display a high level of emotional exhaustion. In a similar vein, prior findings have shown that repeated exposure to customer incivility leads to work stress and emotional exhaustion (see References [18,34,41,43,44]), which provides the evidence that customer incivility is a serious work stressor for service employees. Reference [34] claimed that the negative effects of customer incivility on service employees are more severe than those of coworker incivility because service employees have less leeway in terms of how to deal with and withdraw from uncivil customers than from coworkers.

Second, in the COR framework, positive relationships with customers (e.g., customers’ appreciation and compliments) function as a resource that help service employees regain work-related energy and maintain positive emotions [24]. For instance, service employees restore their resources when their service is appreciated by customers [24]. However, uncivil behavior instigated by customers thwarts this expectation for appreciation, thereby depleting service employees’ resources [24]. In this case, service employees lack resources that are needed for coping with job insecurity, thus easily experiencing emotional exhaustion. As such, customer incivility is a major interpersonal stressor that places emotional demands on service employees and impedes the restoration of their resources. On the basis of COR theory, we proposed that service employees suffer more from job insecurity when exposed to customer incivility. Unlike relationships with coworkers, negative relationships with customers can have a direct impact on the sales performance of service employees [24]. Thus, uncivil customer behavior may render service employees more worried about their job performance and job security, which aggravates and negatively influences perceptions of job insecurity. Furthermore, based on the COR tenet that individuals who lack resources are vulnerable to further resource loss, it is presumed that service employees facing customer incivility are more susceptible to the resource-depleting effects of job insecurity, thereby experiencing more emotional exhaustion. We therefore put forth the following moderation hypothesis:

**Hypothesis** **3.** *The relationship between job insecurity and emotional exhaustion is moderated by customer incivility, such that the positive relationship between job insecurity and emotional exhaustion is more pronounced when customer incivility is high than when it is low.*

## 3. Method

### 3.1. Data Collection Procedure and Sample Characteristics

Our sample consisted of employees in various service organizations (airlines, banks, hospitality, retail, etc.) who were recruited by an online survey company located in South Korea. The online survey company reached a subject pool of 1.2 million South Koreans, who reported their occupation when they registered for online membership through a user authentication system (e.g., cellular phone number or email address). Such online survey panels have been used as a reliable source for access to diverse samples [45,46]. The online survey company allowed us to contact its members who were employed in service organizations and solicit their participation in a study of work and stress (i.e., job insecurity, coworker incivility, and customer incivility). We asked interested individuals to fill out a pre-screen questionnaire to collect their demographic information and email address. Among the individuals who completed the pre-screen questionnaire, we sent a survey invitation to service employees in non-managerial positions whose work experience was longer than one year. The survey invitation stated the voluntary nature of participation, guaranteed the anonymity of responses, and included instructions for the surveys. We emailed the respondents links to the online survey site at two time points. The online survey company monitored data integrity through traps for geo-IP violators and timestamps to flag fast responding, which prohibited respondents from logging into the survey site and filling out the surveys multiple times. Respondents received US $3 as a reward for participation.

We employed a two-wave research design. We chose a time lag of three months between Time 1 (T1) and Time 2 (T2) surveys because work stressors have been found to affect employee work outcomes within a shorter (two- to three-month) rather than a longer (six- to eight-month) time period [47,48,49]. We administered the T1 survey in September 2018 and the T2 survey in December 2018, which coincided with the annual performance evaluation period of Korean companies, thus enhancing the reliability of job performance measurement (see References [49,50]). The T1 survey comprised items assessing job insecurity, emotional exhaustion, coworker incivility, and customer incivility, as well as positive (PA) and negative affectivity (NA) traits and social desirability bias. The T2 survey measured employees’ own job performance. Of the 555 employees who participated in the T1 survey, thirty-three were excluded due to incomplete responses, yielding 522 respondents for T1 (response rate = 94.1%). Of the 522 respondents, 264 completed the T2 survey (response rate = 48.4%). 

The final sample consisted of 264 full-time employees who worked in various service sectors: retail (e.g., department stores and retail stores) (63.6%), tourism/hospitality (e.g., hotels, restaurants, hospitals, and airlines) (28.0%), and banking/insurance (8.3%). This proportion is representative of the composition of our target population. Fifty-seven percent of the participants were female. The average age of the participants was 36.4 (*SD* = 9.0) years, ranging from 21 to 54 years. The majority of the participants had a four-year university education (53.0%), followed by a two-year college education (30.1%), a graduate-level education (3.0%), and a high school education (23.9%). The participants, on average, reported 4.9 (*SD* = 4.7) years of work experience. 

### 3.2. Measurement Scales

As the original survey items were written in English and translated into Korean. They were then back-translated and validated by bilingual scholars [51]. Except for coworker and customer incivility, all the other variables were measured on a five-point Likert-type scale (1 = ‘‘strongly disagree’’ and 5 = ‘‘strongly agree’’).

Job insecurity was assessed with four items from De Witte’s and Schreurs et al.’s scales [29,52]. An example of the items was, “I think that I will lose my job in the near future” (*α* = 0.81). 

Emotional exhaustion was measured using four items of Maslach and Jackson’s scale [26]. A sample item was, “I feel emotionally drained from my work” (*α* = 0.79). 

To assess job performance, we used four items from Williams and Anderson’s and Way et al.’s scales [53,54]. An example of the items was, “I adequately completed my assigned duties” (*α* = 0.89). 

To measure coworker incivility, the participants were asked to report the extent to which they experienced incivility from coworkers in the workplace. We used a four-item scale [24] and response options ranged from 1 (never) to 5 (very often). Sample items included, “How often does your coworker ignore or exclude you while at work?” and “How often is your coworker rude to you at work?” (*α* = 0.89).

Customer incivility was measured with nine items from Wilson and Holmvall’s scale [55]. Examples of the items were, “How often have customers continued to complain despite your efforts to assist them?” and “How often have customers made gestures (e.g., eye rolling, sighing) to express their impatience?” (*α* = 0.93).

We controlled for the participants’ age, gender, education, and organizational tenure in all subsequent analyses due to the potential confounding effects on emotional exhaustion (see References [49,56]) and job performance (see References [57,58,59,60]). In addition, because our surveys dealt with sensitive issues (e.g., job insecurity, coworker incivility, and customer incivility), we needed to include social desirability bias as a control [61]. Social desirability bias was assessed with five items from Hays et al.’s short social desirability bias scale [62]. Sample items included, “I am always courteous, even to people who are disagreeable” and “There have been occasions when I took advantage of someone” (*α* = 0.86). Finally, consistent with Reference [57], we controlled for positive affectivity (PA) and negative affectivity (NA) by using six items from the International Positive Affect and Negative Affect Schedule Short Form [63] (PA—active, attentive, and inspired; α = 0.89; NA—upset, hostile, and nervous; α = 0.81).

## 4. Results

### 4.1. Test of Reliability, Validity, and Common Method Variance (CMV)

Table 1 presents the means, standard deviations, Cronbach’s alpha coefficients, and correlations of the study variables. The Cronbach’s alphas for the scales ranged from 0.81 to 0.93, which demonstrates a high level of reliability [64]. To evaluate the convergent and discriminant validity, we performed a confirmatory factor analysis (CFA) with the M-plus 8.2 program. The proposed eight factor model (i.e., job insecurity, coworker incivility, customer incivility, emotional exhaustion, job performance, social desirability bias, PA, and NA) exhibited a good fit in an absolute sense (*χ*^2^_(566)_ = 998.60; *p* < 0.05; CFI (comparative fit index) = 0.92; TLI (Tucker Lewis index) = 0.92; RMSEA (root mean square error of approximation) = 0.05; and SRMR (standardized root mean square residual) = 0.05). Furthermore, the eight constructs displayed a sufficient level of composite reliability, ranging from 0.81 to 0.93 (see Table 1). Additionally, we assessed the discriminant validity among the constructs based on Fornell and Larcker’s procedure [65]. Table 1 shows that all AVEs (average variance extracted) were larger than the squared correlation between the target construct and any of the other constructs.

Because we relied on self-reported measures, we explored the possibility that the participants’ responses were affected by common method variance (CMV). According to Reference [66], researchers can control CMV with statistical and procedural remedies. Based on their recommendation, we employed procedural remedies by protecting respondent anonymity, reducing evaluation apprehension, improving item wording, and separating the measurement of the predictor and outcome variables. Moreover, we performed Harman’s one-factor analysis as a statistical remedy [66]. All measures of the goodness of fit indicated a worse fit for the one-factor model than for the original measurement model (*χ*^2^_(594)_ = 4374.37; *p* < 0.05, CFI = 0.34, TLI = 0.30, RMSEA = 0.16, SRMR = 0.17). Finally, we used an additional latent common method factor (LCMF) in which every item in the baseline model was allowed to load (in addition to loading on its respective construct). LCMF accounted for 10.56% of the total variance, which was considerably less than the median method variance (25%) found in research using self-reported data [67]. These findings suggest that CMV was not a serious threat to the empirical rigor of our analyses.

### 4.2. Hypothesis Testing

We tested our hypotheses in two steps. First, we examined a simple mediation model to test Hypothesis 1. Second, to test the moderation hypotheses (Hypotheses 2 and 3) and moderated mediation effects (post-hoc analysis), we conducted moderated mediation analyses. Prior to the main analyses, all continuous variables were mean-centered [68]. To estimate the mediation, moderation, and moderated mediation effects, we used an M-plus macro designed by Hayes and Stride et al. [69,70]. Hypothesis 1 proposed a mediating effect of emotional exhaustion on the relationship between job insecurity and job performance. We tested this hypothesis through a bootstrapping (*N* = 5000) procedure, a statistical resampling method that estimates the standard deviation of a model from a sample [69]. The results showed that, controlling for gender, age, education, organizational tenure, social desirability bias, PA, and NA, the indirect effect of job insecurity on job performance through emotional exhaustion was significant (*b* = −0.046, 95% CI = [−0.080, −0.022]). Moreover, when emotional exhaustion was included in the model, the direct effect of job insecurity on job performance was still statistically significant (*b* = −0.102, 95% CI = [−0.181, −0.027]), suggesting a partial mediation (see Table 2). Therefore, Hypothesis 1 was supported.

**Figure 1:**
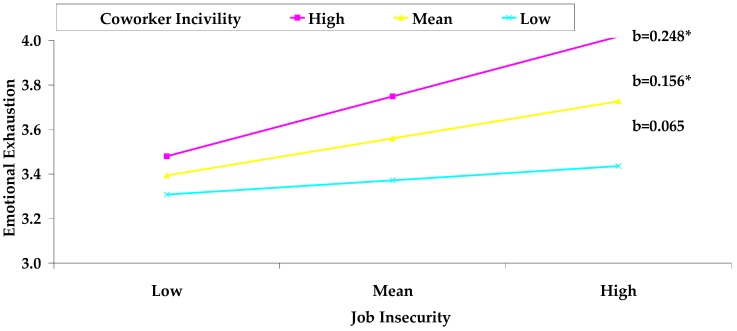
The moderating effect of coworker incivility on the job insecurity–emotional exhaustion relationship; Note: * *p* < 0.05.

Hypothesis 2 postulated the moderating effect of coworker incivility on the relationship between job insecurity and emotional exhaustion. We tested this hypothesis by conducting moderation analyses, which accounted for the possibility that statistically significant indirect effects are contingent on the value of the proposed moderating variable [69]. As shown in Table 3, coworker incivility strengthened the positive relationship between job insecurity and emotional exhaustion (*b* = 0.12, *p* < 0.05). In addition, as depicted in Table 4 and Figure 1, a follow-up simple slope analysis (plotting simple slopes at ±1 *SD* of the moderator) demonstrated that the positive relationship between job insecurity and emotional exhaustion was more pronounced among employees with high and mean levels of coworker incivility (high: *b* = 0.248, 95% CI = [0.095, 0.404]; mean: *b* = 0.156, 95% CI = [0.054, 0.250]). In contrast, job insecurity was not associated with emotional exhaustion when employees perceived a low level of coworker incivility (low: *b* = 0.065, 95% CI = [−0.044, 0.176]). These findings lend support to Hypothesis 2.

Hypothesis 3 predicted that customer incivility would have a moderating effect on the job insecurity–emotional exhaustion relationship. We did not detect any significant moderating effects of customer incivility (*b* = 0.01, *p* = n.s.). Therefore, Hypothesis 3 was not supported.

### 4.3. Post-Hoc Analysis

**Figure 2:**
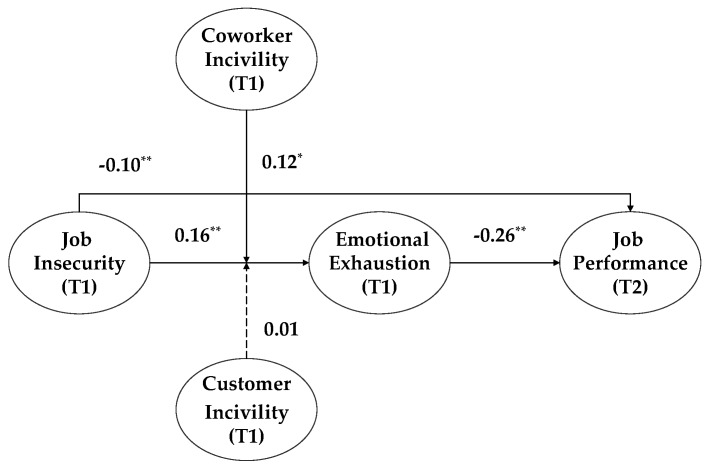
Moderated mediation model. Note: * *p* < 0.05, ** *p* < 0.01.

As Hypotheses 1 and 2 were supported, we further tested a potential moderated mediation model in which the indirect effects of job insecurity on job performance through emotional exhaustion were contingent upon the level of coworker incivility (see Figure 2). We found a significant moderated mediation effect (*b* = −0.029, 95% CI = [−0.066, −0.001]) (see Table 3). Table 4 further illustrates that the indirect effect of job insecurity on job performance through emotional exhaustion was significant for high and mean levels of coworker incivility (high: *b* = −0.064, 95% CI = [−0.117, −0.024]; mean: *b* = −0.040, 95% CI = [−0.072, −0.015]). On the other hand, when coworker incivility was low, the indirect effect of job insecurity on job performance through emotional exhaustion was not significant (low: *b* = −0.017, 95% CI = [−0.048, 0.011]). 

## 5. Discussion

The purpose of this study was to test the mediating effect of emotional exhaustion on the relationship between service employees’ job insecurity and performance, as well as the moderating effects of coworker incivility and customer incivility on the job insecurity–emotional exhaustion link. As predicted, service employees’ job insecurity had a significant positive relationship with their job performance three months later through the intermediary process of emotional exhaustion. Of the two forms of incivility, only coworker incivility moderated the relationship between job insecurity and emotional exhaustion. Our post-hoc analysis revealed that coworker incivility moderated not only the job insecurity–emotional exhaustion relationship but also the indirect effect of job insecurity on job performance through emotional exhaustion. These findings have implications for theory and practice.

### 5.1. Theoretical Implications

While a vast amount of research has investigated the individual and organizational consequences of job insecurity, relatively little attention has been paid to the role of job insecurity in service contexts or boundary conditions that strengthen or weaken the negative effects of service employees’ job insecurity. The results of our mediation analysis replicate prior findings denoting a positive association between job insecurity and emotional exhaustion (see References [33,71]) as well as a mediated relationship between job insecurity, emotional exhaustion, and job performance (see Reference [32]). Our findings demonstrated that job insecurity was strong enough to exert a long-term negative effect on the job performance of service employees. They further revealed that, similar to other occupations, emotional processes are a key intervening mechanism through which service employees’ job insecurity impairs their job performance. However, distinct from prior research that proposed and tested the effect of job insecurity as generalizable to different occupations, our study aimed to develop a model of job insecurity addressing the effect of job insecurity and its boundary conditions in the service industry. A notable strength of our research design was the recruitment of participants from several organizations in the service sector, which enhanced the generalizability of the study findings to the entire service industry. By uncovering how the job performance of job-insecure service employees was reduced and when this negative effect was aggravated, our study offers insights into how to manage the job insecurity of service workers.

Our findings make a theoretical contribution to the job insecurity literature by adopting AET and COR theory as overarching theoretical frameworks. Our mediation and moderation analyses generally supported AET and COR theory. As predicted by AET, our research identified a negative affective response (i.e., emotional exhaustion) as a central mediator that transmitted the negative effects of job insecurity to job performance. Moreover, by demonstrating the moderating role of coworker incivility, our findings endorsed the AET proposition that work contexts can facilitate the effects of a workplace event on affective responses. The findings of our study further revealed that the negative impact of workplace events (i.e., job insecurity) on affective responses could be boosted in the presence of a negative work context (i.e., coworker incivility), which validated the premise that individuals are more sensitive to negative work events and overestimate the occurrences of those events [38]. The present findings also corroborated the findings of COR theory, which suggests that job insecurity is a major work stressor that drains the emotional resources necessary for task accomplishment. In support of the COR framework that suggests the long-term resource-depleting effect of work stressors [12], the findings of the present study indicated that job insecurity exerted a long-term deleterious effect on job performance by draining emotional resources. In addition, our findings endorsed the loss spiral of COR theory, which suggests that a loss of resources or inadequate restoration of those resources renders individuals vulnerable to further loss of resources [12]. In line with this theorizing, our findings showed that service employees whose resources are depleted due to job insecurity become susceptible to the loss of social resources, become more emotionally exhausted, and perform poorly when exposed to coworker incivility. Thus, our study extended current theoretical explanations of job insecurity by validating AET and COR as pertinent theoretical frameworks that address the job insecurity of service employees.

Our study also provided a comprehensive understanding of the role of interpersonal relationships in job insecurity by demonstrating coworker incivility as a boundary condition of job insecurity. Job insecurity research adopting the COR framework has identified social support as a moderator that buffers the negative impact of job insecurity on employee outcomes [14,15,16]. This line of research assumes support (e.g., work-related assistance and emotional support) from coworkers or supervisors as social resources that help employees cope with work stressors. While prior research offers fruitful insight into interpersonal factors that mitigate the negative effects of job insecurity, interpersonal factors that aggravate the negative effect of job insecurity remain unknown in the literature. The present study is a preliminary attempt to reveal such interpersonal factors. Our findings indicate that the negative effects of job insecurity become worse when employees experience uncivil behavior from their coworkers. Complementing prior findings that highlight the role of social support in job-insecurity situations, our findings suggested that not only providing adequate social support but also preventing or reducing coworker incivility is a way to help employees cope with job insecurity. 

Contrary to our prediction, customer incivility failed to moderate the relationship between job insecurity and emotional exhaustion, which contradicts the findings of Reference [34] that posit that customer incivility is more strongly related to personal and organizational outcomes than is coworker incivility. The stronger effect of coworker than customer incivility may be because we examined the interaction between job insecurity and incivility. The job insecurity literature suggests that intra-group relations or social exchange processes strongly affect perceptions of job insecurity [72]. Coworkers are influential sources that shape employees’ perceptions and interpretations of workplace events. In particular, in situations of job-insecurity, coworkers provide important information for coping [16]. Thus, uncivil behaviors perpetrated by coworkers tend to strongly affect employees’ emotional reactions in job-insecurity [73]. Another explanation for the stronger effect of coworker incivility in our data pertains to the retrospective nature of our surveys. Because we instructed respondents to provide retrospective ratings of uncivil behaviors they had experienced, they were likely to more easily recall uncivil behaviors instigated by coworkers who were more familiar to them than customers, which might have caused the effect of coworker incivility to be stronger than that of customer incivility. Scholars have reported that in service jobs involving frequent and close interactions with customers (e.g., post office workers), the deleterious effect of customer incivility was as strong as that of coworker incivility (see References [74,75]). Therefore, the relative importance of coworker and customer incivility needs to be disentangled in future investigations into the effects of different forms of incivility in different service contexts and jobs.

### 5.2. Practical Implications

Our findings offer practitioners ideas for managing job insecurity in service organizations. Based on the present findings indicating that job insecurity is a key work stressor causing emotional exhaustion and impaired performance in service employees, freeing employees from concerns about job security would be the best solution to tackle job-insecurity issues. However, as job insecurity is an inevitable phenomenon in today’s organizations [2], it is unrealistic to remove all threats to job security. Yet, scholars suggest some feasible ways to mitigate employees’ perceptions of job insecurity. First, given that job insecurity stems from uncertainty about job continuity [2], job insecurity perceptions can be weakened by articulating performance criteria and expectations [16]. Another way to reduce perceptions of job insecurity is by providing employees with interventions or training suited for boosting their confidence and efficacy and motivating them to set challenging but achievable performance goals [1]. 

It is also critical that organizational leaders develop a precise understanding of conditions that can exacerbate the negative impacts of job insecurity on employee outcomes. In our study, coworker incivility turned out to be such a boundary condition. As coworker incivility is prevalent in the workplace without being noticed or reported, organizational leaders are advised to prevent and reduce coworker incivility by institutionalizing organizational policies and procedures to monitor and punish uncivil behavior in the workplace [49,76]. Organizations may also need to consider devising training programs to heighten employees’ awareness of the perpetration and consequences of coworker incivility [77]. In those programs, organizational leaders or human resource managers can demonstrate examples of uncivil behaviors at work and their potential impacts as well as desirable work behaviors and manners [18]. In addition, workplace counseling or stress-management interventions are recommended for coping with coworker incivility [43]. Finally, based on the findings that managerial support can mitigate the negative impact of coworker incivility [78], organizational leaders need to play an active role in providing support for the victims and potential targets of coworker incivility.

### 5.3. Limitations and Directions for Future Research

The findings of the current study should be interpreted in light of the following limitations. First, we relied on service employees’ self-reports to evaluate their job performance. However, self-reported measures of job performance are susceptible to social desirability and rater biases. For this reason, organizational researchers recommend the use of other-ratings (e.g., supervisor ratings, peer ratings, and 360-degree feedback) and objective performance (e.g., sales revenue and customer evaluation) as measures of job performance. Future research needs to use those measures to assess job performance more rigorously.

Second, although we employed two-wave surveys to reduce CMV and test a lagged effect of job insecurity, our research design was not longitudinal in a strict sense. As job insecurity and emotional exhaustion were simultaneously measured at T1, the causal relationship between the two variables should be interpreted with caution. The significant association between job insecurity and emotional exhaustion may reflect the possibility that emotionally exhausted employees reported a high level of job insecurity. To establish causality between job insecurity, emotional exhaustion, and job performance, a three-wave longitudinal design would be desirable for future research. 

Third, while our findings generally supported AET and COR theory, there was an omission in our moderated mediation model that warrants further empirical investigations. Both AET and COR theory posit that individuals’ reactions to work stressors are contingent upon their individual characteristics. AET identifies personality traits as such individual characteristics [11]. Similarly, COR theory maintains that employees’ personal resources affect how they deal with job demands or work stressors. That is, individuals with adequate personal resources have a greater reservoir of resources that help them cope with stressful events. Therefore, for a complete understanding of job insecurity through AET and COR lenses, it is necessary to delve into the moderating effect of personal resources such as self-efficacy or resilience on the relationship between job insecurity and employee outcomes.

Fourth, unlike many other forms of counterproductive work behavior, the intensity of incivility can escalate over time in an ongoing interaction between the target and the instigator [73]. Thus, the single measurement of coworker and customer incivility may not capture the multiple, spiraling nature of workplace incivility. We recommend that future research explore the temporal dynamics of workplace incivility on employee outcomes. In addition, although we only focused on coworker and customer incivility, scholars have noted that individuals of higher organizational status tend to be more frequent instigators of uncivil behavior than those of lower status [73], which leaves supervisor incivility as a future research topic. While we confined our sample to lower-rank employees in the present study, the job level of incivility instigators and targets should be taken into account for a nuanced understanding of the effects of different forms of workplace incivility. 

Finally, we acknowledge that our sample only consisted of Korean service employees. Due to a continuing downturn in the Korean economy, Korean employees are facing a job-insecurity crisis (i.e., employment rate of 66.6% in 2018) [79], which may have caused the strong perceptions of job insecurity in our data. Furthermore, since Korean culture is characterized by a high level of collectivism [80], relationships with coworkers are deemed important in the Korean workplace. For this reason, coworker incivility may have exerted a stronger moderating effect for Korean service employees than customer incivility. It should also be noted that the use of online surveys may have resulted in a sampling bias, as suggested by the large proportion of young employees in our sample [81]. Therefore, future research needs to validate the current findings in samples that represent different cultures and age groups. 

## Figures and Tables

**Table 1:** Means, standard deviations, and correlations.

Variables	M	SD	α	CR	1	2	3	4	5	6	7	8	9	10	11	12
1. Gender	0.43	0.50	-	-	-											
2. Age	36.42	8.96	-	-	0.13 *	-										
3. Education	14.70	1.75	-	-	0.11 ^†^	−0.11 ^†^	-									
4. Job Tenure	4.93	4.72	-	-	0.05	0.42 **	−0.06	-								
5. Social Desirability Bias	3.47	0.58	0.86	0.86	0.05	0.06	0.02	−0.02	0.55							
6. PA	2.65	0.91	0.89	0.89	0.06	0.10	0.04	0.02	0.17 **	0.74						
7. NA	2.79	0.91	0.81	0.81	−0.11 ^†^	−0.18 *	0.01	0.00	−0.05	−0.24 **	0.60					
8. Job Insecurity	2.84	1.04	0.91	0.91	0.01	−0.01	−0.01	−0.00	0.00	−0.18 ^**^	0.32 **	0.71				
9. Emotional Exhaustion	2.37	0.78	0.79	0.80	0.04	−0.00	−0.11 ^†^	0.05	−0.25 ^**^	−0.18 ^**^	0.36 **	0.34 **	0.51			
10. Job Performance	4.03	0.60	0.88	0.88	−0.11 ^†^	−0.03	−0.01	−0.11 ^†^	0.31 **	0.11 ^†^	−0.07	−0.25 **	−0.42 **	0.64		
11. Coworker Incivility	2.12	0.82	0.89	0.89	0.04	0.06	−0.04	−0.00	−0.09	−0.06	0.30 **	0.25 **	0.40 **	−0.21 **	0.68	
12. Customer Incivility	2.64	0.82	0.93	0.93	−0.08	−0.22 *	−0.04	−0.04	0.02	−0.14 ^*^	0.48 **	0.22 **	0.21 **	0.05	0.30 **	0.61

**Notes:**^†^*p* < 0.10, * *p* < 0.05, ** *p* < 0.01. Numbers along the diagonal are the AVEs (average variance extracted). CR—composite reliability; PA—positive affectivity; NA—negative affectivity.

**Table 2:** Test of the mediation of emotional exhaustion.

Path	Effect *(b)*	95% CI_low_	95% CI_high_
Indirect Effect			
Job Insecurity → Emotional Exhaustion → Job Performance	−0.046	−0.080	−0.020
Direct Effect			
Job Insecurity → Job Performance	−0.102	−0.181	−0.027
Total Effect			
Job Insecurity → Job Performance	−0.148	−0.226	−0.075

**Table 3:** Test of the moderating effects of coworker and customer incivility.

Variables	Emotional Exhaustion			Job Performance		
	*b*	se	*t*	*B*	Se	*t*
Gender	0.09	0.08	1.06	−0.10	0.07	1.53
Age	0.00	0.00	0.17	0.00	0.00	0.24
Education	−0.04	0.02	1.88	−0.02	0.02	0.84
Job Tenure	0.01	0.01	0.66	−0.01	0.01	1.62
Social Desirability Bias	−0.29	0.07	4.22	0.26	0.06	4.46
PA	−0.06	0.05	1.26	0.01	0.04	0.16
NA	0.17	0.05	3.27	0.07	0.04	1.82
Job Insecurity	0.16	0.04	3.81	−0.10	0.03	3.03
Coworker Incivility	0.23	0.05	4.30			
Customer Incivility	−0.00	0.06	0.02			
Emotional Exhaustion × Coworker Incivility	0.12	0.05	2.45			
Emotional Exhaustion × Customer Incivility	0.01	0.05	0.17			
Emotional Exhaustion				−0.26	0.05	5.45
*R* ^2^	28.3%			26.3%		
*Moderated Mediation Index*						
Emotional Exhaustion × Coworker Incivility → Emotional Exhaustion → Job Performance: *b* = −0.029, 95% CI = [−0.066, −0.001]						
Emotional Exhaustion × Customer Incivility → Emotional Exhaustion → Job Performance: *b* = −0.002, 95% CI = [−0.030, 0.019]						

Note: * *p* < 0.05.

**Table 4:** Test of the conditional effect of job insecurity on job performance through emotional exhaustion.

Path	Job Insecurity Emotional Exhaustion				Job Insecurity Emotional Exhaustion Job Performance			
Moderators	Level	*b*	Cl_95%low_	Cl_95%high_	Level	*b*	Cl_95%low_	Cl_95%high_
Coworker Incivility	1.30 (−1 *SD*)	0.065	−0.044	0.176	1.30 (−1 *SD*)	−0.017	−0.048	0.011
	2.12 (Mean)	0.156	0.054	0.250	2.12 (Mean)	−0.040	−0.072	−0.015
	2.94 (+1 *SD*)	0.248	0.095	0.404	2.94 (+1 *SD*)	−0.064	−0.117	−0.024
Customer Incivility	1.82 (−1 *SD*)	0.150	0.035	0.277	1.82 (−1 *SD*)	−0.039	−0.078	−0.010
	2.64 (Mean)	0.156	0.054	0.250	2.64 (Mean)	−0.040	−0.072	−0.015
	3.46 (+1 *SD*)	0.162	0.031	0.298	3.46 (+1 *SD*)	−0.042	−0.085	−0.008

Note: ^*^ p < 0.05. CI—confidence interval; *b*—unstandardized coefficient.
